# Integrated Serum Pharmacochemistry, Metabolomics, and Network Pharmacology to Reveal the Material Basis and Mechanism of Danggui Shaoyao San in the Treatment of Primary Dysmenorrhea

**DOI:** 10.3389/fphar.2022.942955

**Published:** 2022-07-11

**Authors:** Hui Xiong, Na Li, Lanqingqing Zhao, Zhe Li, Yongzhou Yu, Xiaoyan Cui, Qi Liu, Chunying Zhao

**Affiliations:** ^1^ Hebei Province Key Laboratory of Study and Exploitation of Chinese Medicine, Chengde Medical University, Chengde, China; ^2^ Hebei Key Laboratory of Nerve Injury and Repair, Chengde Medical University, Chengde, China; ^3^ Institute of Basic Medicine, Chengde Medical University, Chengde, China; ^4^ Hebei Institute for Drug and Medical Device Control, Shijiazhuang, China; ^5^ The Research Institute of Medicine and Pharmacy, Qiqihar Medical University, Qiqihar, China

**Keywords:** Danggui Shaoyao San, material basis and mechanism, serum pharmacochemistry, metabolomics, network pharmacology

## Abstract

Danggui Shaoyao San (DSS), a well-known formula, has been successfully applied in treating primary dysmenorrhea (PD) in China. However, its material basis and mechanism are still unrevealed. This current research aims to reveal the material basis and mechanism of DSS in treating PD by an integrative approach of serum pharmacochemistry, metabolomics, and network pharmacology. The results showed that DSS markedly relieved the physiological and pathological symptoms of PD as confirmed by the improvement of writhing behavior, inhibition of uterine edema, callback of clinical biochemical indexes, and metabolic profiles. Furthermore, a metabolomic analysis demonstrated that the therapeutic effect of DSS was attributed to the modulation of arachidonic acid metabolism, pentose and glucuronate interconversions, and phenylalanine metabolism. Meanwhile, 23 blood ingredients were identified after the oral administration of DSS. By analyzing the correlation coefficient of the identified biomarkers and blood components, active compounds closely associated with core metabolic pathways were extracted. Taking these active compounds as a basis, network pharmacology prediction was executed. It was found that active components of DSS including alisol B,23-acetate, chlorogenic acid, levistilide A, cianidanol, senkyunolide A, atractylenolide II, and sedanolide, were germane to steroid hormone biosynthesis, arachidonic acid metabolism, sphingolipid signaling pathway, etc. Interestingly, PTGS2 and PTGS1 related to the arachidonic acid metabolism may be pivotal targets of DSS. The current study proved that the integration of serum pharmacochemistry, metabolomics, and network pharmacology, was a powerful approach to investigate the material basis and the molecular mechanisms of DSS, and provided a solid basis for DSS application.

## Introduction

Primary dysmenorrhea (PD) refers to periodic lower abdominal pain and non-organic lesions in women during menstruation or before and after menstruation (Osuga et al., 2020; Karout et al., 2021). PD is one of the most common gynaecological disorders, with a prevalence rate of 60%–90% in young women and 15% in severe cases, which has become one of the major events in the normal work and study of women (Azagew et al., 2020). Previous research found that PD appeared to be a consequence of uterine myometrium hyperactivity and uterine ischemia occurrence induced by excessive release of prostaglandins, and ovarian hormones, such as cervical factors, vasopressin, etc. (Fang et al., 2017; Chai et al., 2020; Martone et al., 2021). At present, the treatment methods of PD include drug therapy and supplementary drug therapy. Non-steroidal anti-inflammatory drugs and oral contraceptives are the most commonly used drugs to relieve pain by inhibiting the excessive accumulation of prostaglandins. Nevertheless, their long-term use can produce many gastrointestinal side effects (Marjoribanks et al., 2015; Vahedi et al., 2021). Considering the complexity of PD pathogenesis, it requires the coordinated treatment of non-toxic side-effects and multi-flavored drugs. Danggui Shaoyao San (DSS) is recognized as a well-known formula for the clinical treatment of PD with a long clinical practice in China (Lee et al., 2016). It consists of *Angelica sinensis* (Oliv.) Diels, *Paeonia lactiflora* Pall., *Ligusticum chuanxiong* Hort., *Poria cocos* (Schw.) Wolf, *Atractylodes macrocephala* Koidz., and *Alisma plantago-aquatica* Linn. Modern medical research shows that DSS has a variety of beneficial effects, such as inhibiting uterine contraction (Hua et al., 2008), regulating vasomotor substances (Tzeng et al., 2013), improving blood supply (Kim et al., 2016), and improving the anti-inflammatory (Yin et al., 2016) and immune regulations (Bi et al., 2021). Through reliable clinical and animal studies, it was ascertained that DSS treatment on PD exhibited a significant analgesic effect via regulating ovarian hormone secretion, vasorelaxation, and inflammatory reactions (Zhou et al., 2015). Scholars have reported that the material basis of DSS against atherosclerosis was paeoniflorin, ferulic acid, and Poria acid, and the material basis of DSS against senile dementia are ferulic acid, *A. macrocephala* I, gallic acid, etc. (Wu et al., 2020; Bi et al., 2021). Nevertheless, for hundreds of years, due to the complexity of the components and internal mechanisms of DSS, tremendous efforts have been made to reveal the active components and pharmacological mechanisms of DSS, but it still has not been effectively solved.

With the continuous innovation of research theory and technology in recent years, the technical systems of serum pharmacochemistry, network pharmacology, and metabolomics guided by the characteristics of overall regulation and dialectical treatment of traditional Chinese medicine (TCM) have become a hotspot (Chen et al., 2016; Schrimpe-Rutledge et al., 2016; Gao et al., 2021). The system pharmacology methods of material basis and action mechanism of TCM are diverse, and each technology has its own advantages. Integrating various methods for an all-round research is one of the main problems faced at present (He et al., 2021). Serum pharmacochemistry can reflect the body’s effect on drug absorption and metabolism and the interaction of drugs in the body, and the false-positive or false-negative phenomenon caused by the blind separation of chemical components *in vitro* was effectively avoided (Wang et al., 2021). It is the most material-based screening method in line with human pharmacokinetics, but it does not involve the study of its functional mechanism. However, network pharmacology was used to successfully construct a complex biological network of “disease–gene–target–drug” interaction through massive database resources and research tools, and make virtual predictions of functional mechanisms during drug therapy, so as to make up for the deficiency of serum pharmacochemistry (Huang et al., 2020; Liu et al., 2021; Nogales et al., 2022). However, network pharmacology is a virtual screening platform driven by big data, which are not strongly related to the real condition of drug action *in vivo*. Therefore, it can be verified with the help of action targets and pathways determined by high-throughput metabolomic technology.

In this work, we proposed a novel approach that serum pharmacochemistry, network pharmacology, and metabolomics were integrated to investigate the material basis and mechanisms of DSS for the treatment of PD. Firstly, the therapeutic mechanisms of DSS on PD by analyzing writhing behavior, histopathology of uterine, clinical biochemical indexes, and urine metabolic profiles was confirmed. Subsequently, blood ingredients of DSS in the PD model were identified by serum pharmacochemistry. A correlation analysis of the blood compounds and biomarkers was conducted to identify the core bioactive compounds and metabolomic pathways. Taking the screened active compounds as a basis, a network pharmacology prediction was then performed to investigate the molecular mechanisms. Finally, integrating the results of the network pharmacology prediction and metabolomics, the core active components, targets, and metabolic pathways were screened out. This study gives prominence to the feasibility and availability of a powerful strategy-based serum pharmacochemistry, network pharmacology, and metabolomics that provide a meaningful insight into the material basis and mechanisms of DSS in treating PD.

## Materials and Methods

### Chemicals and Reagents

Six crude drugs, Angelicae Sinensis Radix [Apiaceae, *A. sinensis* (Oliv.) Diels, Lot number: C2592012003], Paeoniae Radix Alba (Buttercup, *P. lactiflora* Pall., Lot number: C2232012004), Chuanxiong Rhizoma (Apiaceae, *L. chuanxiong* Hort., Lot number: C2472012004), Poria [Polyporaceae, *P. cocos* (Schw.) Wolf, Lot number: C0452104001], Atractylodis Macrocephalae Rhizoma (Asteraceae, *A. macrocephala* Koidz., Lot number: C2242012003), and Alismatis Rhizoma (Alismataceae, *A. plantago-aquatica* Linn., Lot number: C3862012002), were provided from Hebei Hehuachi Pharmaceutical Co., Ltd. (Chengde, China) and identified by Professor Zhanhui Su of Hebei Key Laboratory of Study and Exploitation of Chinese Medicine, Chengde Medical College. Acetonitrile, ethanol, and methanol were obtained from Fisher Scientific Corporation (United States). ELISA kit for β-endorphin (β-EP), prostaglandin F_2α_ (PGF_2α_), prostaglandin E_2_ (PGE_2_) and estradiol (E_2_), progesterone (Prog), and endothelin (ET) were provided from Nanjing Jiancheng Biotechnology Co. Ltd. (Nanjing, China).

### Danggui Shaoyao San Sample Preparation

According to the ancient records from “Synopsis of Golden Chamber,” *A. sinensis* (Oliv.) Diels, *P. lactiflora* Pall., *L. chuanxiong* Hort., *P. cocos* (Schw.) Wolf, *A. macrocephala* Koidz., and *A. plantago-aquatica* Linn., were made into powder crushed to 50 mesh, and weighed at the ratio of 3, 16, 8, 4, 8, and 4 g, respectively. The mixture was refluxed for 2 h with 430 ml of 50% ethanol for the first time, and then refluxed for 1 h with 344 ml of 50% ethanol for the second time. The combined filtrate was dried under vacuum freeze-drying conditions to obtain a freeze-dried powder of its ethanol extract. The freeze-dried powder of DSS was dissolved with distilled water to obtain the DSS samples (0.02 g/ml) for the HPLC analysis and the intragastric solution (0.33 g/ml).

### HPLC Conditions

The chromatographic separation was performed on an Agilent 1220 Liquid chromatograph (Palo Alto, United States) equipped with a ZORBAX SB-C18 column (4.6 mm × 250 mm, 5 μm). The elution system contained mobile phase acetonitrile (A) and 0.2% formic acid water (B). The elution procedure is as follows: 0–44 min, 5%–27% (A); 44–54 min, 27%–51% (A); 54–90 min, 51%–78% (A). The detection wavelength was 300 nm, the detector column’s temperature was 30°C, the sample volume was 10 μl, and the flow rate was 1.0 ml/min. The chromatogram of DSS is shown in Supplementary Figure S1.

### Animals and Treatments

Forty specific pathogen-free (SPF) grade female Sprague–Dawley rats (174 ± 10 g) were obtained by Huafukang Biotechnology Co., Ltd. (Beijing, China). The rats were maintained at a standard laboratory environment with the temperature of 25°C ± 3°C, humidity 60% ± 5%, a 12 h alternating day and night, and had free access to water and a standard diet. After acclimatization in a metabolic cage for 7 days, all animals were stochastically split into four groups (10 rats/group): control group (CON), model group (MOD), ibuprofen-positive drug group (POS), and Danggui Shaoyao San treatment group (DSS). Apart from the CON group, rats from the remaining three groups were injected subcutaneously with estradiol benzoate (2 mg/ml) once daily at a dose of 1.0 mg/kg on day 1 and day 10, and 0.5 mg/kg from day 2 to day 9. Half an hour after the subcutaneous injection of estradiol benzoate on day 10, oxytocin (20 U/mL) was injected intraperitoneally at a dose of 25 U/kg. The CON group was injected with the same volume of saline solution. According to four times and one time of clinical equivalent dose, the DSS group and POS group were administered with the DSS solution (0.33 g/ml, dissolved in distilled water) and ibuprofen solution (5.4 mg/ml, dissolved in 0.50% CMC-Na) at a dose of 10 ml/kg once daily from the first day of modeling for 7 consecutive days, respectively. Meanwhile, the CON and MOD groups were administered with the same volume of distilled water. All operations were strictly subject to the Ethical Committee of Chengde Medical University (IACUC Issue Number:CDMULAC-20210906-014).

### Uterine Indices and Behavioral Analysis

Estrogen stimulates the uterus to make it hyperemia and edema, resulting in an abnormal uterine and body weight, as well as uterine dysfunction in rats. Therefore, the feasibility of the estrogen-induced dysmenorrhea model can be judged by uterine index and body weight (Nordéus et al., 2012). According to the characteristics of abdominal contraction concave, trunk and hind limb extension, hip and lateral limb internal rotation of rats, the writhing times, and the writhing latency of rats were observed and calculated within 30 min after oxytocin injection. Then, the uterus index was computed as follows: Uterus indices = uterus mass/body mass.

### Biochemical Parameters and Histopathology Analysis

We determined the contents of β-EP, PGF_2α_, and PGE_2_ in the uterine tissue and the contents of E_2_, Prog, and ET in the serum by using an ELISA kit. The uterine sections were stained with hematoxylin and eosin, then observed and photographed under the visible light microscope, and the images were analyzed by Case Viewer 2.3 software (3DHistech Ltd.).

### Serum and Urine Samples’ Preparation

Abdominal aortic whole blood was collected 60 min after oral administration on the 10th day. Blood samples were centrifuged at 3,500 rpm for 10 min at 4°C to acquire serum samples. Firstly, 2 ml of 4% phosphoric acid solution was mixed with 2 ml of the serum sample. After a normatively ultrasonic and vortexing treatment, the mixed solution was applied to a pre-activated OASIS HLB solid phase extraction (SPE) C18 column. Then, the SPE column was washed successively with 2 ml water and 2 ml 100% methanol. The methanol eluent was collected, and dehydrated under vacuum conditions at 40°C. The dried samples were mixed with 400 μl of 60% methanol, sonicated for 30 s, and vortexed for 30 s, and then centrifuged at 13,000 rpm for 10 min at 4°C. 5 μl of the supernatant was applied for *in vivo* component. Urine samples were collected on day 10 of administration. 200 μl urine sample and 600 μl distilled water were mixed vigorously for 30 s, and centrifuged at 13,000 rpm, 4°C for 10 min. 2 μl of the supernatant was injected for a urine metabolomic analysis. A collection of all the samples was prepared as the quality control (QC) sample.

### UPLC-MS Conditions of Urine Metabolomics and Component Identification

Urine and blood samples were separated for the global analysis on an ACQUITY UPLC BEH C18 (100 mm × 2.1 mm i.d., 1.7 μm; Waters Corporation, Milford, CT, United States) using an LC-30A UPLC system (Shimadzu, Japan). The column temperature and the flow rate were preset at 40°C and 0.4 ml/min, respectively. The elution gradient conditions for urine metabolomics and component identification *in vivo* are provided in Supplementary Tables S1, S2, respectively. The optimal mass spectrometry parameters were as follows: atomization pressure (GS I) 55 Psi, auxiliary pressure (GS II) 55 Psi, air curtain pressure 35 Psi, ion source temperature 500°C, Scanning range 100–1,500 Da, collision energy −40 eV or 40 eV, and spray voltage −4500 V or 5500 V (negative and positive ion mode). To ensure accurate mass readings, an APCI calibration solution as the reference was performed for monitoring ions in two ion modes.

### Data Processing and Multivariate Data Analysis

All the data preprocessed by MarkerView 1.3.1 software were inputted into Ezinfo 3.0 software for the multivariate data analysis. PCA and OPLS-DA was used to identify the differential variables across the groups based on VIP value greater than 1 and the relative intensity difference of the variables between the groups (*p* < 0.05). Subsequently, the fragment information in databases such as HMDB, METLIN databases combined with MasterView 1.2 software accommodated in the PeakView 2.2 workstation was applied to identify the substances.

### Correlation Analysis of Urine Biomarkers and Absorbed Components

To unearth the material basis in DSS that actually acts a critical role in PD treatment, the relative intensity of absorbed components and urine biomarkers were introduced into the metaboanalyst 5.0 software for the correlation analysis. Following the screening principle that the correlation coefficient of blood components is greater than 0.6, it is regarded as a highly relevant material basis and contributes greatly to the therapeutic effect.

### Material Basis–Target Prediction

TCMSP (http://tcmspw.com/tcmsp.php), a platform for systems pharmacology and compound ingredient database, was applied to screen the targets of material basis. Genecards (https://www.genecards.org) and the OMIM database (http://www.omim.org/) were used to search targets of PD. Their common targets are used to predict metabolic pathways. The integrated analysis of the potential pathway results of network pharmacology and metabolomics was performed to find the core mechanism of material basis.

### Statistical Analysis

Data are expressed as the mean ± SD for the statistical analysis of data between two groups by SPSS version 25.0. Using Student’s t-test, a value of *p* less than 0.05 was regarded statistically significant for all analyses.

## Results

### General Characteristics, Uterine Indices, and Behavioral Analysis

**FIGURE 1:**
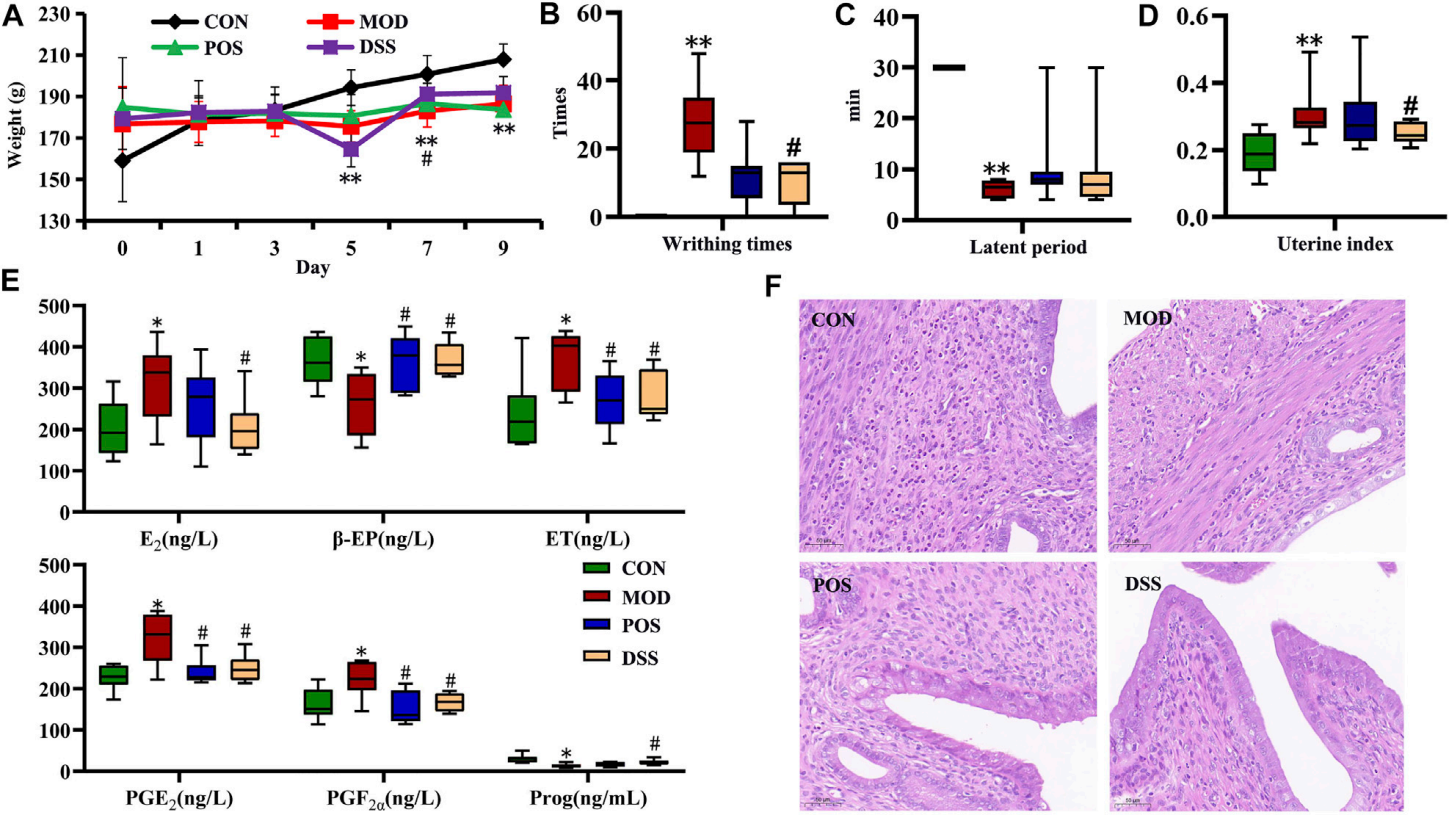
Effect of DSS intervention on PD based on general characteristics, behavioral manifestations, biochemical levels, and pathological changes. **(A)** Body weight, **(B)** writhing times, **(C)** latent period of writhing response, **(D)** uterine index, **(E)** biochemical parameters in serum (E_2_, ET, and Prog), and uterine (PGE_2_, PGF_2α_, and β-EP), **(F)** representative micrographs of uterine histopathology at magnification ×100. Data are presented as the mean ± SD; *p < 0.05, **p < 0.01 vs. CON group, ^#^ *p* < 0.05, and ^##^ *p* < 0.01 vs. MOD group.

The results of body weight, uterine index, and writhing response are shown in Figure 1. Compared with the CON group, the body weight of each administration group and model group increased slowly, and decreased significantly on day 5 (*p* < 0.01). After administration intervention, the weight loss of the DSS group was inhibited (*p* < 0.05) (Figure 1A). After oxytocin injection, writhing times and latent period in the MOD group were significantly increased (*p* < 0.01). After treatment with ibuprofen or DSS, the writhing times of rats in each administration group were significantly decreased (*p* < 0.05) (Figures 1B,C). Compared with the CON group, the uterine index in the MOD group was markedly increased (*p* < 0.01). After treatment, each administration group showed a certain callback with a significant difference (*p* < 0.05) (Figure 1D). All these results not only showed that the rat model of PD was successfully established, but also implied that DSS has a therapeutic effect on PD.

### Biochemical Parameters and Histopathology Analysis

Compared with the CON group, the levels of serum Prog in the MOD group were markedly decreased (*p* < 0.05), and the levels of E_2_ and ET were markedly increased (*p* < 0.05) (Figure 1E). Meanwhile, the levels of PGF_2α_ and PGE_2_ in rat uterine homogenate were significantly increased (*p* < 0.05), and the levels of β-EP were significantly decreased (*p* < 0.05). Compared with the MOD group, the POS group and DSS group showed a certain callback (*p* < 0.05) (Figure 1E). No significant difference between the two administration groups was found, indicating that DSS has a therapeutic effect on PD, which was equivalent to that of positive drugs. Pathological results showed that large-scale endometrial exfoliation and severe edema were found in the lamina propria, with more vacuoles, loose cytoplasm, and light staining in the edema area in the MOD group. In the POS and DSS groups, the endometrium showed less exfoliation, mild edema, decreased vacuoles in the edema area, and a relatively dense cytoplasm (Figure 1F).

### Multivariate Data Analysis

**FIGURE 2:**
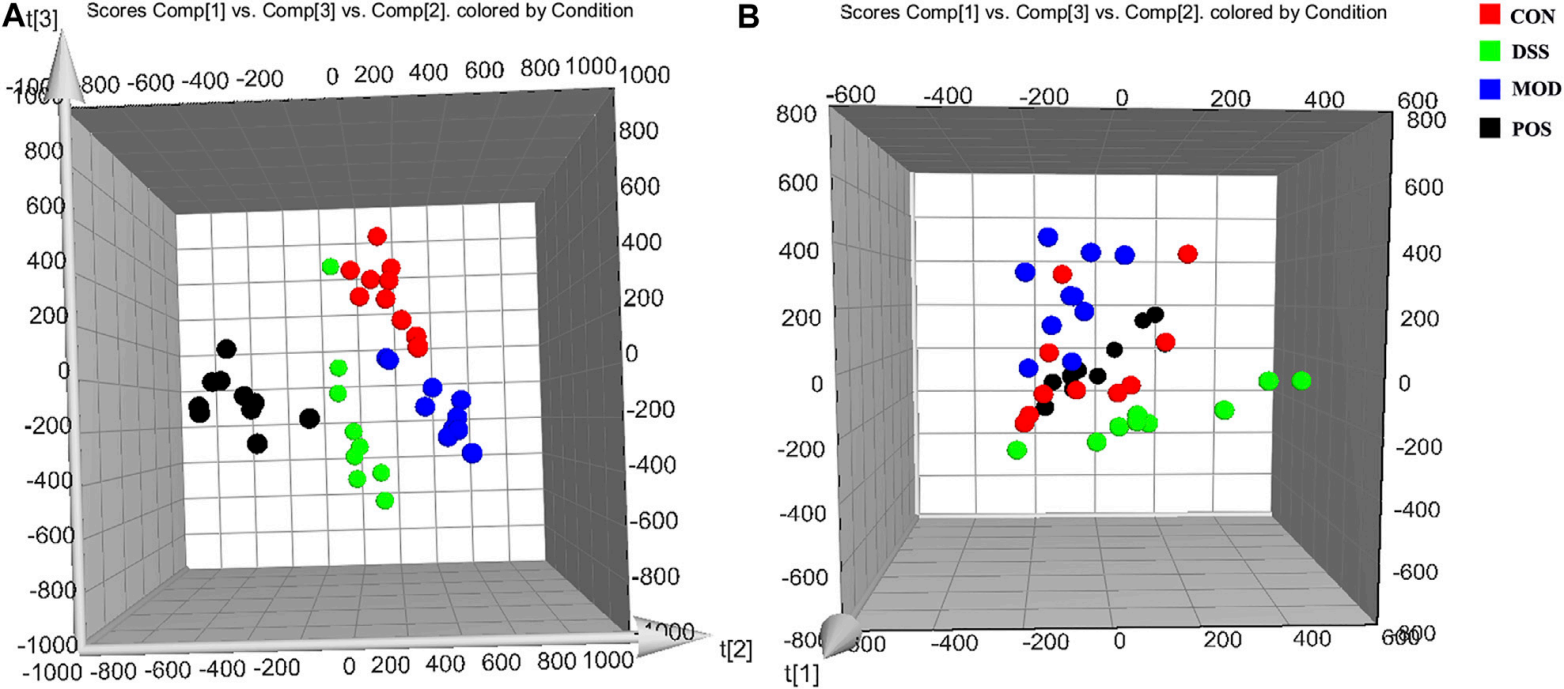
3D PCA score plot of the urine samples in each group on the 10th day generated by Ezinfo 3.0 software. **(A)** Negative ion mode. **(B)** Positive ion mode.

According to the previously established UPLC-MS analysis methods, the urine sample data of the rats on the 10th day CON group, MOD group, POS group, and DSS group were collected. The PCA score plot was obtained by using the multivariate data analysis, and the metabolic profiles of rats in each group were compared and analyzed, as shown in Figure 2. On the 10th day of modeling, the CON group was significantly separated from the MOD group, which further proved that the rat model was successfully replicated. Compared with the MOD group, each administration group could callback the metabolic profile close to the normal group and away from the MOD group. Once again, it proves the effectiveness of DSS in the intervention of PD.

### Differential Biomarkers and Metabolic Pathway Identification

**FIGURE 3:**
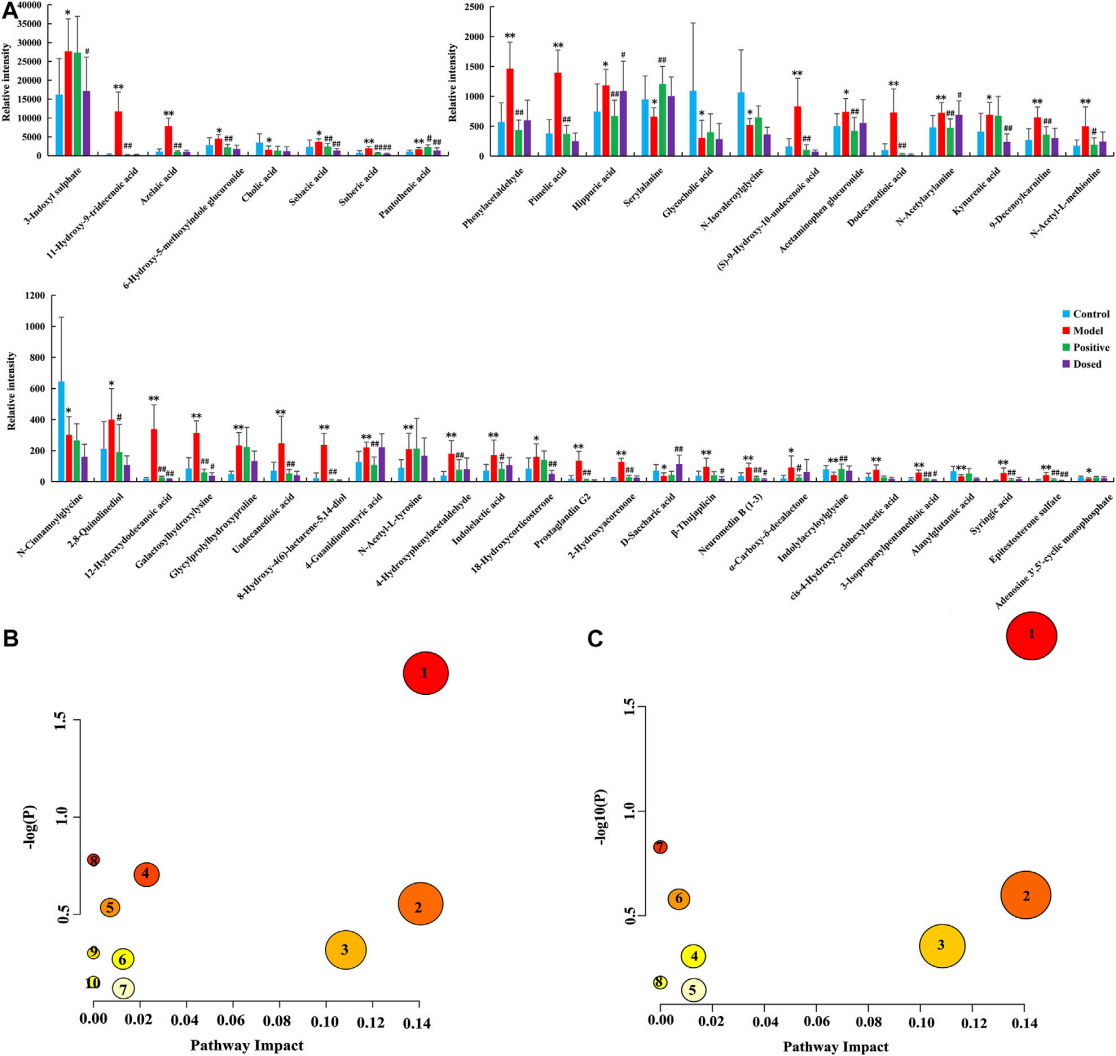
Systems analysis of the metabolomic alterations in each group. **(A)** Histogram of the relative expression levels of the biomarkers, **(B)** Metabolic pathway analysis associated with the pathogenesis of PD by MetaboAnalyst 5.0 (1. phenylalanine metabolism, 2. pentose and glucuronate interconversions, 3. arachidonic acid metabolism, 4. primary bile acid biosynthesis, 5. pantothenate and CoA biosynthesis, 6. tyrosine metabolism, 7. steroid hormone biosynthesis, 8. ascorbate and aldarate metabolism, 9. arginine and proline metabolism, and 10. purine metabolism), **(C)** Metabolic pathway analysis associated with the efficacy of DSS by MetaboAnalyst 5.0 (1. phenylalanine metabolism, 2. pentose and glucuronate interconversions, 3. arachidonic acid metabolism, 4. tyrosine metabolism, 5. steroid hormone biosynthesis, 6. pantothenate and CoA biosynthesis, 7. ascorbate and aldarate metabolism, and 8. purine metabolism).

VIP plot was obtained through an OPLS-DA analysis between the CON group and the MOD group. The differences between the groups were analyzed by using the *t*-test, and the ions with VIP > 1 and *p* < 0.05 were selected as candidate variables. The secondary cleavage information of the candidate variables under certain collision energy was accurately collected and analyzed according to the cleavage law of mass spectrometry, HMDB, ChemSpider databases, and references. Referring to the aforementioned identification model, the chemical structures of 46 potential biomarkers were identified. The results are presented in Supplementary Table S3. Moreover, the histogram (Figure 3A) were generated to analyze the content level of the identified biomarkers in each group. It was found that among the 46 biomarkers in the urine of primary dysmenorrheal model rats, DSS had a callback effect on 42 of them, and 12 of them were statistically significant, including 3-indoxyl sulphate, epitestosterone sulfate, sebacic acid, linoleic acid, pantothenic acid, hippuric acid, etc.

To further classify the metabolic pathways related to PD, the names, HMDB codes, and molecular formulas of the 46 metabolic biomarkers were introduced into the metaboanalyst 5.0 software to obtain 10 metabolic pathways, mainly including phenylalanine metabolism, arachidonic acid metabolism, primary bile acid biosynthesis, pentose and glucuronate interconversions, pantothenate and CoA biosynthesis, tyrosine metabolism, steroid hormone biosynthesis, ascorbate and aldarate metabolism, arginine and proline metabolism, and purine metabolism (Figure 3B). Then, in order to classify the efficacy targets of DSS, the 42 biomarkers recalled were introduced into the metaboanalyst 5.0 software to obtain 8 metabolic pathways, mainly including pentose and glucuronate interconversions, arachidonic acid metabolism, phenylalanine metabolism, tyrosine metabolism, steroid hormone biosynthesis, pantothenate and CoA biosynthesis, ascorbate and aldarate metabolisms, and purine metabolism (Figure 3C). Based on the screening criteria of the pathway impact value greater than 0.05 and literature reports, three metabolic pathways closely associated with PD and drug efficacy including arachidonic acid metabolism, pentose and glucuronate interconversions, and phenylalanine metabolism were obtained.

### Absorbed Component Analysis

**FIGURE 4:**
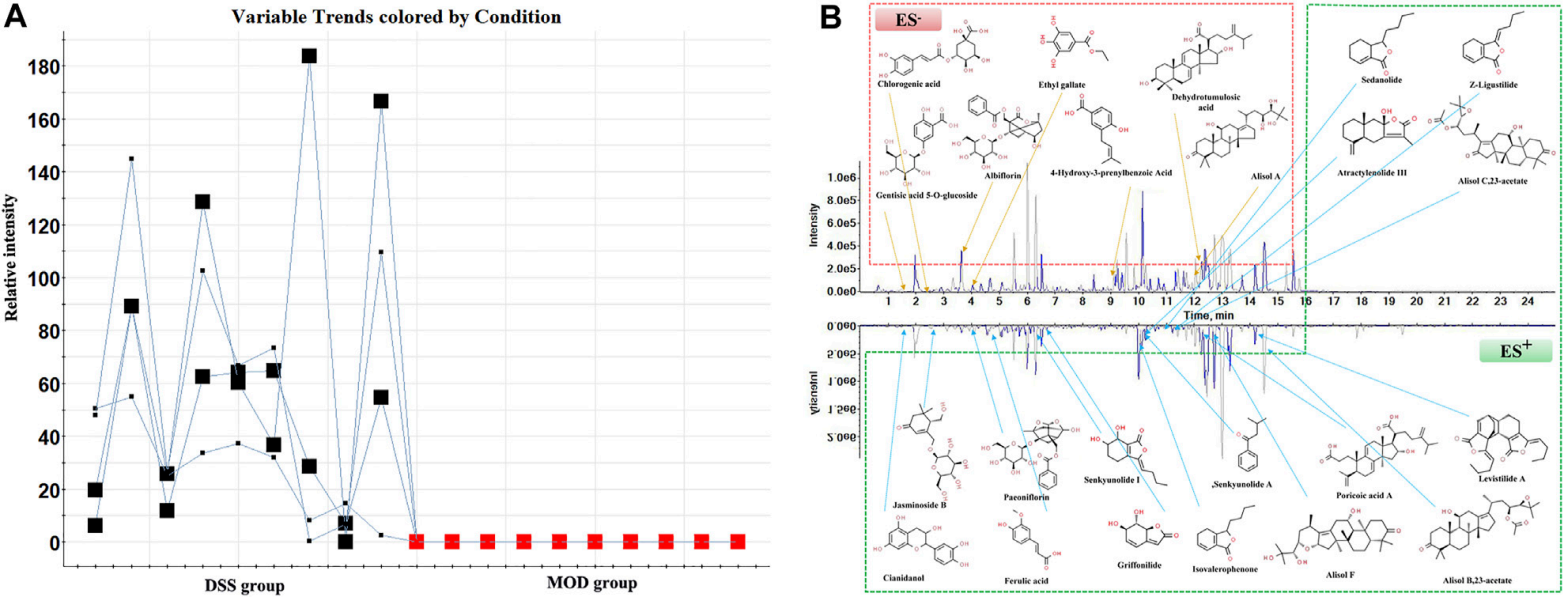
Analysis of the constituents absorbed into the blood from DSS. **(A)** The trend plot of relative intensity of the candidate variables in the MOD and DSS groups, **(B)** UPLC-MS chromatograms and chemical structures of the constituents absorbed into the blood from DSS in the ESI^+^ and ESI^−^ modes.

Due to the low content of blood components in rats, the data alignment, normalization, and matching in the component database including HMDB, Chemspider were carried out with the help of MarkerView 1.3.1 software. Combined with Ezinfo 3.0 software and the previously identified chemical composition of DSS (Li et al., 2022), the trend plots of candidate variables were obtained to discover the ions only existing in the DSS group, and finally, 23 blood components were identified (Figure 4A). The detailed results are provided in Supplementary Table S4. Among them, alisol B acetate, poricoic acid A, alisol F, alisol C 23-acetate, and alisol A were classified as triterpenoids. Levistilide A, sedanolide, Z-Ligustilide, and 3-N-butyl-4,5-dihydrophthalide were classified as butylphthalides. Dehydrotumulosic acid, 4-Hydroxy-3-prenylbenzoic acid, ferulic acid, and chlorogenic acid, were classified as organic acids. Atractylenolide III, senkyunolide I, and griffonilide were classified as lactones. Isovalerophenone, cianidanol, albiflorin, and paeoniflorin were classified as flavonoids. Hydroquinones gentisic acid 5-O-glucoside, alkaloid jasminoside B, and polyphenol ethyl gallate were also observed. Meanwhile, 10 originated from *P. lactiflora* Pall., 13 originated from *A. sinensis* (Oliv.) Diels, 8 originated from *A. macrocephala* Koidz., 8 originated from *P. cocos* (Schw.) Wolf, 4 originated from *A. plantago-aquatica* Linn, and 11 originated from *L. chuanxiong* Hort. (Figure 4B).

### Material Basis Identification

**FIGURE 5:**
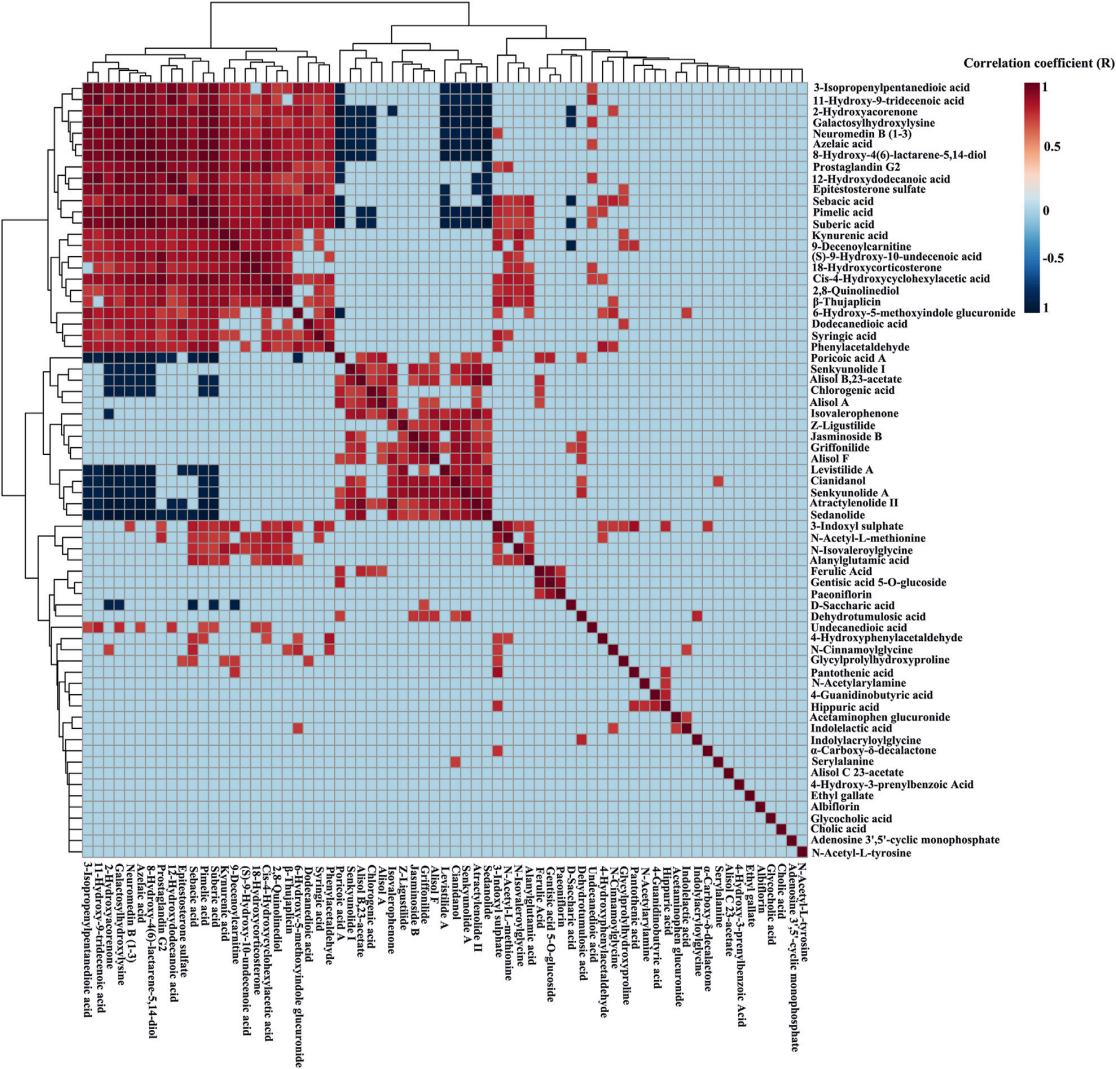
Correlation heatmap between the serum constituents of DSS and the potential biomarkers of PD based on the metaboanalyst 5.0 platform. Heatmaps were drawn based on the relative level of the serum components and potential biomarkers.

The relative content data of the blood components and biomarkers of each rat were imported into the statistical analysis module of the metaboanalyst 5.0 platform, and the absolute threshold of the correlation coefficient (R) was set to 0.7, that was, 0.7 < R ≤ 1, was considered to be extremely correlated, and a correlation heatmap was generated (Figure 5). The number of extreme correlations between candidate blood components and biomarkers reached 5 or more, which can be regarded as material basis. A total of 9 extremely correlated components including poricoic acid A, senkyunolide I, alisol B,23-acetate, chlorogenic acid, levistilide A, cianidanol, senkyunolide A, atractylenolide II, and sedanolide, were found to be potential material basis.

### Potential Target Prediction of Material Basis

**FIGURE 6:**
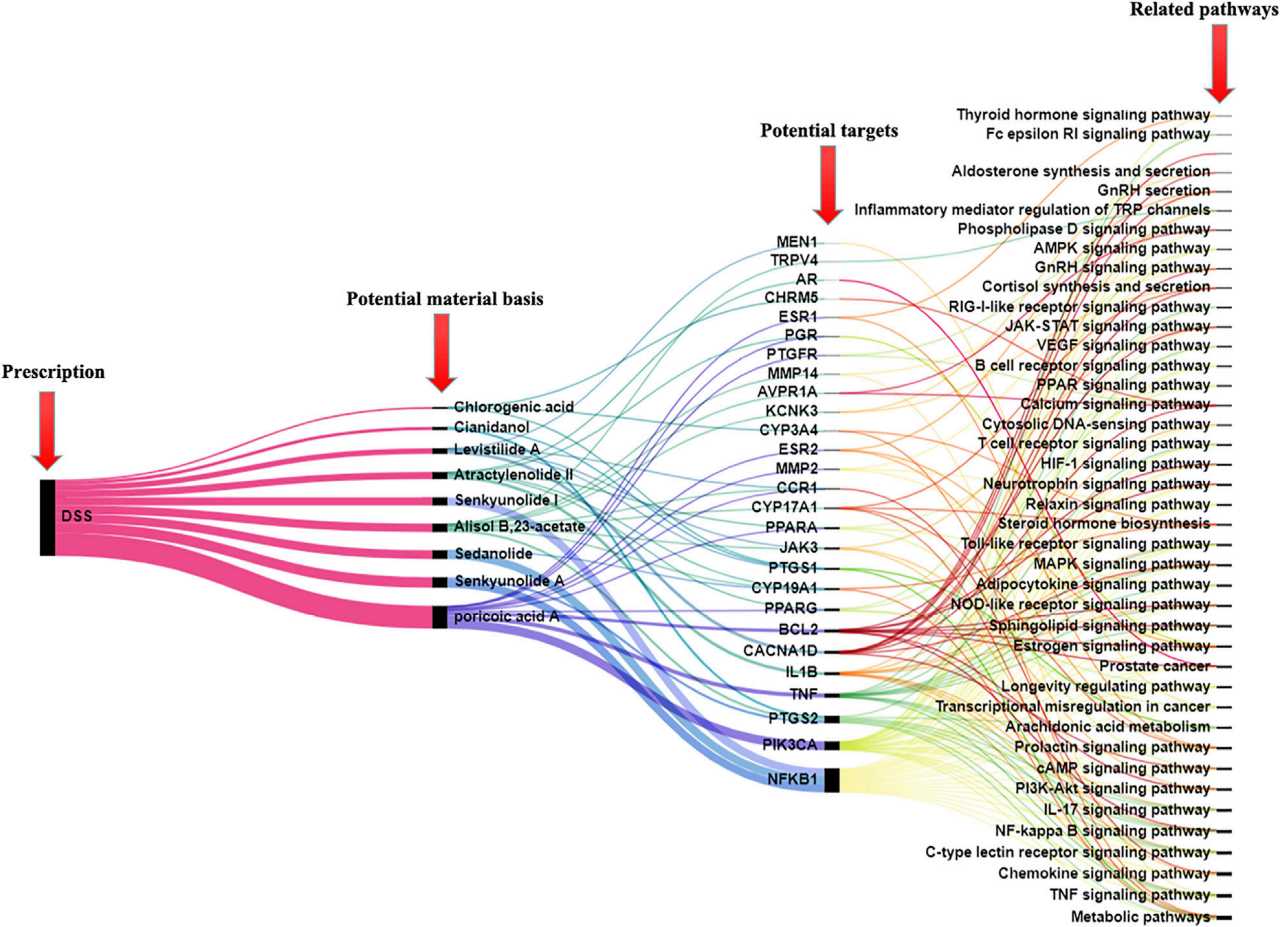
Connection diagram of prescription–potential material basis–potential targets-related pathways. The line thickness represents the degree of connection.

The serum pharmacochemistry theory of TCM believes that only components in blood have the probability to become potential bioactive components after an oral administration of TCM, which can provide a powerful means for the effective identification of active compounds *in vivo* in TCM (Wang et al., 2021). Thus, 9 components screened from serum components of TCM and most related to the endogenous metabolism of the disease are more meaningful as the research object of the follow-up network pharmacological analysis. In order to confirm the effects of these components, they were introduced into TCMSP to predict targets. Then, genecards and the OMIM database were used to search PD-related targets and obtain the intersection targets. There were 27 common targets in total, including Cytochrome P450 3A4 (CYP3A4), Steroid 17-alpha-hydroxylase/17,20 lyase (CYP17A1), tumor necrosis factor (TNF), phosphatidylinositol 4,5-bisphosphate 3-kinase catalytic subunit alpha isoform (PIK3CA), prostaglandin G/H synthase 2 (PTGS2), prostaglandin G/H synthase 1 (PTGS1), aromatase (CYP19A1), etc. Through the enrichment analysis of the common target KEGG pathway (prediction by false discovery rate < 0.05), these potential targets were considered to be involved in 40 pathways, including steroid hormone biosynthesis, VEGF signaling pathway, estrogen signaling pathway, IL-17 signaling pathway, arachidonic acid metabolism, sphingolipid signaling pathway, cortisol synthesis and secretion, thyroid hormone signaling pathway, etc (Figure 6; Supplementary Table S5). Integrating the metabolic pathway results of metabolomics, it was worth noting that two common pathways including steroid hormone biosynthesis related to CYP3A4, CYP17A1, CYP19A1, and arachidonic acid metabolism related to PTGS1, PTGS2 were closely associated with the mechanism of DSS, which meant that alisol B,23-acetate, chlorogenic acid, levistilide A, cianidanol, senkyunolide A, atractylenolide II, and sedanolide were material basis for the DSS treatment on PD.

## Discussion

As an effective preparation for the treatment of PD, the main components of DSS, such as chlorogenic acid, levistilide A, senkyunolide A, have shown good therapeutic effects in PD, uterine bleeding, and inflammatory activities (Saltan et al., 2016; Chen et al., 2021; Zhang et al., 2021). Through the analysis of body weight, uterine organ index, and writhing behavior, it was found that the body weight of rats recovered to a certain extent, and the uterine index, writhing latency, and writhing times could be significantly improved after the continuous administration of DSS for 10 days. Biochemical index results showed that the expression level of pain factors such as E_2_, ET, Prog, β-EP, PGF_2α_, and PGE_2_ had different degrees of the callback effect after administration intervention. Furthermore, uterine morphological changes also showed that DSS could significantly inhibit endometrial exfoliation and edema in PD rats. From the perspective of metabolomics, it was found that DSS exerted its efficacy by modulating arachidonic acid metabolism, phenylalanine metabolism, pentose and glucuronate interconversions, and adjusting the metabolic trajectory of prostaglandin G2, hippuric acid, 6-hydroxy-5-methoxyindoglucuronide, etc. which confirmed the effectiveness of DSS on PD.

The fundamental mechanism of PD was considered to be related to the secretion of endometrial prostaglandins (PGs). Prostaglandins are a kind of unsaturated fatty acids, which widely exist in various important organs and tissues such as the female endometrium and follicles (Wrobel and Mlynarczuk, 2018). Prostaglandin G2 (PGG2) is produced by the conversion of arachidonic acid through the action of cyclooxygenase. Overexpression of PGG2 will promote the generation of prostaglandin E2 (PGE_2_) and prostaglandin F_2α_ (PGF_2α_). The production of PGE_2_ and PGF_2α_ represented a key node in prostaglandin metabolism, which participated in the regulation of relaxation and contraction of uterine smooth muscle (Li WJ et al., 2021). During menstruation, the endometrium of patients with PD secreted a large amount of PGs, which was significantly higher than that of normal women. It was mainly due to the decrease of progesterone secretion and the release of arachidonic acid before menstruation in patients with dysmenorrhea, which was oxidized to PGs under the action of cyclooxygenase. When prostaglandin secretion is excessive, it will induce the contraction of blood vessels and myometrium, resulting in ischemia and pain (You et al., 2021). In addition, increasing prostaglandin levels may also improve the perception of peripheral neuralgia (Cruz et al., 2012). In this current study, we found that the content of PGG2 in MOD rats was higher than that in CON rats, indicating that the arachidonic acid metabolism remained abnormal, which promoted the contraction of the uterine smooth muscle, and eventually led to the occurrence of dysmenorrhea. DSS could partially repair the arachidonic acid metabolism as evinced by the inhibition of PGG2, which could reduce the synthesis of PGF_2α_ and PGE_2_ expressions to alleviate pain symptoms.

It was reported that inflammatory response and oxidative stress were the key mechanisms of the pathogenesis of PD (Augoulea et al., 2009). Due to the decrease in the blood flow to the myometrium during the onset of PD, ischemia will be induced during uterine contraction, which can trigger the accumulation of reactive oxygen free radicals, resulting in the change of thenendometrial cell function, uterine oxidative stress injury, and inflammatory response (Kaplan et al., 2013). Phenylalanine metabolism was generally considered to be involved in oxidative stress and inflammatory response (Horn et al., 2021), while hippuric acid and phenylacetaldehyde were intermediates of the phenylalanine metabolism. Hippuric acid was transformed into acylglycines with the participation of glycine and benzoic acid. Excessive secretion of acylglycine induced mitochondrial fatty acids *β* oxidation disorder and then participated in the inflammatory response (Lin et al., 2013). In addition, the expression level of hippuric acid increased in varying degrees when inflammation occurred (Li et al., 2017). Phenylacetaldehyde was transformed from Phenylpyruvate metabolism. The increase of phenylacetaldehyde content leaded to the abnormal metabolism of phenylalanine. The accumulation of hippuric acid and phenylacetaldehyde in the body indicates that inflammation interfered with the phenylalanine metabolism. After intragastric administration of DSS, the contents of hippuric acid and phenylacetaldehyde in the urine of the rats were decreased, implying that DSS can intervene in the phenylalanine metabolism by regulating inflammatory response and oxidative stress after DSS treatment, and then play a therapeutic role in dysmenorrhea. 6-hydroxy-5-methoxyindole glucuroside was a metabolite of 6-hydroxy-5-methoxyindole produced by uridine diphosphate glucosidase in the liver. Studies have shown that pentose and glucuronate interconversions are involved in the pathogenesis of PD (Huang et al., 2016). 6-hydroxy-5-methoxyindole glucuroside, as an intermediate product of the mutual transformation of pentose and glucuronate, and its abnormal expression may be involved in the pathogenesis of PD. The abnormal expression of the two metabolites of hippuric acid and 6-hydroxy-5-methoxyindole glucuroside during the process of modeling showed that the occurrence and development of PD was accompanied by the disorders of pentose and glucuronate interconversions and phenylalanine metabolism in varying degrees. After treatment, DSS could partly improve the pentose and glucuronate interconversions and phenylalanine metabolism as evinced by the decreasing 6-hydroxy-5-methoxyindole glucuroside level.

In general, this study used serum pharmacochemistry coupled with metabolomic technology to explore the effects of DSS on PD-related biomarkers for unveiling the material basis of DSS. Also, the general behavior, histopathology, and biochemical characteristics confirmed the overall therapeutic effect of DSS and the internal essential connection between PD and DSS. Finally, the potential targets of the material basis were authenticated to assure that the identified potential material basis had to do with the action mechanism of DSS. These findings indicate that 46 biomarkers related to arachidonic acid metabolism, phenylalanine metabolism, and the mutual transformation of pentose and glucuronic acid and so on were identified, and the metabolic pathways related to the efficacy of DSS were traced. The potential material basis of DSS included poricoic acid A, senkyunolide I, alisol B,23-acetate, chlorogenic acid, levistilide A, cianidanol, senkyunolide A, atractylenolide II, and sedanolide extracted by the serum pharmacochemistry and the metabolomic correlation analysis. Taking these pharmacodynamic substances as the research object, further network pharmacological predictions showed that the potential targets that were implemented were mainly involved in steroid hormone biosynthesis, VEGF signaling pathway, estrogen signaling pathway, IL-17 signaling pathway, arachidonic acid metabolism, sphingolipid signaling pathway, cortisol synthesis and secretion, thyroid hormone signaling pathway, etc. Most notably, PTGS2 and PTGS1 related to arachidonic acid metabolism may be pivotal targets of DSS because they appeared in metabolomics and network pharmacology. Based on this, a total of 7 chemical compounds closely related to multiple key targets, such as alisol B,23-acetate, chlorogenic acid, levistilide A, cianidanol, senkyunolide A, atractylenolide II, and sedanolide, were considered as material basis of DSS for the treatment of PD. Of course, the related metabolic pathways of these compounds need to be followed-up in-depth with point-to-point verification. Metabolomics, serum pharmacochemistry, and network pharmacology could exhibit an efficient way to investigate and screen the potential effective compounds and action mechanisms of TCM.

## Conclusion

In present study, an integrative serum pharmacochemistry, metabolomics, and network pharmacology-based strategy was proposed to uncover the material basis and mechanisms of DSS in treating PD. A total of 46 potential biomarkers related to 10 metabolic pathways were clarified, and 23 blood ingredients after oral administration of DSS were identified. Further correlation analysis between serum pharmacochemistry and metabolomics found that a total of 9 extremely correlated components were the potential material basis. Integrating the network pharmacology prediction based on these potential material basis, PTGS2 and PTGS1 related to arachidonic acid metabolism might be pivotal targets of DSS. Meanwhile, we have identified 7 chemical compounds related to multiple key targets, such as alisol B,23-acetate, chlorogenic acid, levistilide A, cianidanol, senkyunolide A, atractylenolide II, and sedanolide, as material basis of DSS for the treatment of PD. These results illustrate that serum pharmacochemistry, metabolomics, and network pharmacology strategy supply a powerful approach for exploring the material basis and underlying mechanisms of TCM.

## Data Availability

The original contributions presented in the study are included in the article/Supplementary Material, further inquiries can be directed to the corresponding authors.
