# Supplementary material for: Integrated Serum Pharmacochemistry, Metabolomics, and Network Pharmacology to Reveal the Material Basis and Mechanism of Danggui Shaoyao San in the Treatment of Primary Dysmenorrhea
Source: Front Pharmacol. 2022 Jul 11;13:942955. doi: 10.3389/fphar.2022.942955 (PMC9310033; doi:10.3389/fphar.2022.942955)
Supplement: Supplementary file 1 [file DataSheet1.docx]

**TABLE S1** The optimal UPLC linear gradients of urine metabolomics analysis

| Time ( min ) | Flow rate (mL/min) | Water with 0.1% formic acid | Acetonitrile with 0.1% formic acid | curve |
| --- | --- | --- | --- | --- |
| 0 | 0.4 | 95 | 5 | 6 |
| 3.5 | 0.4 | 85 | 15 | 6 |
| 5.5 | 0.4 | 80 | 20 | 6 |
| 8.5 | 0.4 | 60 | 40 | 6 |
| 9.5 | 0.4 | 0 | 100 | 6 |
| 11.5 | 0.4 | 0 | 100 | 6 |

**TABLE S2** The optimal UPLC linear gradients of component identification in vivo

| Time ( min ) | Flow rate (mL/min) | Water with 0.1% formic acid | Acetonitrile with 0.1% formic acid | curve |
| --- | --- | --- | --- | --- |
| 0 | 0.4 | 5 | 95 | 6 |
| 3.5 | 0.4 | 15 | 85 | 6 |
| 6.5 | 0.4 | 30 | 70 | 6 |
| 12.5 | 0.4 | 70 | 30 | 6 |
| 18.5 | 0.4 | 100 | 0 | 6 |
| 25 | 0.4 | 100 | 0 | 6 |

**TABLE S3** Potential biomarkers and metabolomic pathways identiﬁed of PD model rats in ESI^-^ and ESI^+^ mode based on UPLC-MS

| NO | Rt | m/z | Mass Error (ppm) | Formula | Compound | Fragments | Trend | Metabolomic pathway |
| --- | --- | --- | --- | --- | --- | --- | --- | --- |
| 1 | 0.64 | 209.0303 | 0.3 | C_6_H_10_O_8_ | D-Saccharic acid | 85,59 | ↓ | Ascorbate and aldarate metabolism |
| 2 | 0.66 | 146.0923 | -0.2 | C_5_H_11_N_3_O_2_ | 4-Guanidinobutyric acid | 111,86,56 | ↑ | Arginine and proline metabolism |
| 3 | 0.72 | 259.1653 | -6.2 | C_15_H_24_O_2_ | 2-Hydroxyacorenone | 259,127,84 | ↑ | - |
| 4 | 0.79 | 330.0597 | -0.1 | C_10_H_12_N_5_O_6_P | Adenosine 3',5'-cyclic monophosphate | 136,119 | ↓ | Purine metabolism |
| 5 | 1.14 | 330.1301 | -2.0 | C_12_H_19_N_3_O_5_ | Glycylprolylhydroxyproline | 312,270,211,115 | ↓ | - |
| 6 | 1.48 | 218.1032 | -0.5 | C_9_H_17_NO_5_ | Pantothenic acid | 146,116,99,88 | ↓ | Pantothenate and CoA biosynthesis |
| 7 | 2.11 | 181.0505 | -0.2 | C_8_H_8_O_2_ | 4-Hydroxyphenylacetaldehyde | 163,135,134 | ↑ | Tyrosine metabolism |
| 8 | 2.25 | 222.0771 | -0.2 | C_11_H_13_NO_4_ | N-Acetyl-L-tyrosine | 180,163,107 | ↑ | - |
| 9 | 2.43 | 190.0537 | -3.1 | C_7_H_13_NO_3_S | N-Acetyl-L-methionine | 148,142,98 | ↑ | - |
| 10 | 2.52 | 158.0820 | -1.6 | C_7_H_13_NO_3_ | N-Isovaleroylglycine | 136,77 | ↓ | - |
| 11 | 2.57 | 195.0628 | 0.5 | C_8_H_12_O_4_ | 3-Isopropenylpentanedioic acid | 175,150,108,85 | ↑ | - |
| 12 | 2.60 | 190.0498 | -0.2 | C_10_H_7_NO_3_ | Kynurenic acid | 172,144,116,89 | ↑ | - |
| 13 | 2.68 | 326.0873 | -2.3 | C_14_H_17_NO_8_ | Acetaminophen glucuronide | 175,150,113,108 | ↑ | - |
| 14 | 2.86 | 212.0020 | -0.9 | C_8_H_7_NO_4_S | 3-Indoxyl sulphate | 132,92,79 | ↑ | - |
| 15 | 3.06 | 206.0456 | -1.4 | C_9_H_7_NO_2_ | 2,8-Quinolinediol | 162,147,118 | ↑ | - |
| 16 | 3.16 | 180.0652 | -1.3 | C_9_H_9_NO_3_ | Hippuric acid | 105,77,53 | ↑ | Phenylalanine metabolism |
| 17 | 3.17 | 134.0607 | -3.1 | C_8_H_9_NO | N-Acetylarylamine | 100,77 | ↑ | - |
| 18 | 3.53 | 159.0660 | -1.4 | C_7_H_12_O_4_ | Pimelic acid | 115,97,95 | ↑ | - |
| 19 | 3.76 | 197.0453 | -0.9 | C_9_H_10_O_5_ | Syringic acid | 182,166,123,95 | ↑ | - |
| 20 | 4.40 | 340.1025 | -0.2 | C_15_H_17_NO_8_ | 6-Hydroxy-5-methoxyindole glucuronide | 206,164,122 | ↑ | Pentose and glucuronate interconversions |
| 21 | 4.70 | 203.0925 | 0.1 | C_8_H_14_O_3_ | cis-4-Hydroxycyclohexylacetic acid | 157,141 | ↑ | - |
| 22 | 4.70 | 261.1338 | -2.2 | C_11_H_20_O_4_ | Undecanedioic acid | 243,181,143 | ↑ | - |
| 23 | 5.34 | 165.0553 | -5.9 | C_8_H_8_O | Phenylacetaldehyde | 119,93 | ↑ | Phenylalanine metabolism |
| 24 | 5.40 | 263.0896 | 5.5 | C_8_H_14_N_2_O_5_ | Alanylglutamic acid | 219,203 | ↓ | - |
| 25 | 5.49 | 369.1522 | 2.2 | C_12_H_24_N_2_O_8_ | Galactosylhydroxylysine | 351,195,127 | ↑ | - |
| 26 | 5.49 | 173.0817 | -0.8 | C_8_H_14_O_4_ | Suberic acid | 129,111,109,83 | ↑ | - |
| 27 | 5.54 | 209.0818 | -0.8 | C_10_H_12_O_2_ | β-Thujaplicin | 191,123 | ↑ | - |
| 28 | 5.72 | 204.0662 | -1.9 | C_11_H_11_NO_3_ | Indolelactic acid | 158,130,116 | ↑ | - |
| 29 | 6.31 | 204.0661 | -2.1 | C_11_H_11_NO_3_ | N-Cinnamoylglycine | 160,130,103 | ↑ | - |
| 30 | 6.78 | 187.0971 | -2.3 | C_9_H_16_O_4_ | Azelaic acid | 169,125,97 | ↑ | - |
| 31 | 7.00 | 245.1390 | -2.2 | C_11_H_20_O_3_ | (S)-9-Hydroxy-10-undecenoic acid | 227,209,165 | ↑ | - |
| 32 | 7.20 | 239.1631 | 6.4 | C_12_H_24_O_3_ | 12-Hydroxydodecanoic acid | 221,123,91 | ↑ | - |
| 33 | 7.21 | 273.1700 | -2.9 | C_13_H_24_O_3_ | 11-Hydroxy-9-tridecenoic acid | 255,155 | ↑ | - |
| 34 | 7.23 | 243.0770 | -1.8 | C_13_H_12_N_2_O_3_ | Indolylacryloylglycine | 199,168,142 | ↓ | - |
| 35 | 7.32 | 221.0791 | 6.8 | C_6_H_12_N_2_O_4_ | Serylalanine | 203,175,92 | ↓ | - |
| 36 | 7.41 | 259.1181 | -2.7 | C_11_H_18_O_4_ | α-Carboxy-δ-decalactone | 197,101 | ↑ | - |
| 37 | 7.73 | 201.1129 | -1.5 | C_10_H_18_O_4_ | Sebacic acid | 183,139 | ↑ | - |
| 38 | 8.38 | 229.1442 | -1.2 | C_12_H_22_O_4_ | Dodecanedioic acid | 211,193,149 | ↑ | - |
| 39 | 8.79 | 363.2159 | -1.7 | C_21_H_30_O_5_ | 18-Hydroxycorticosterone | 345,247 | ↑ | Steroid hormone biosynthesis |
| 40 | 8.84 | 369.1720 | -2.6 | C_19_H_28_O_5_S | Epitestosterone sulfate | 289,271 | ↑ | - |
| 41 | 8.99 | 314.2326 | 0.2 | C_17_H_31_NO_4_ | 9-Decenoylcarnitine | 255,157,85 | ↑ | - |
| 42 | 9.58 | 303.1644 | -6.0 | C_12_H_22_N_4_O_5_ | Neuromedin B (1-3) | 243,166 | ↑ | - |
| 43 | 9.58 | 453.2836 | -5.0 | C_24_H_40_O_5_ | Cholic acid | 407,112 | ↓ | Primary bile acid biosynthesis |
| 44 | 9.62 | 466.3154 | -1.7 | C_26_H_43_NO_6_ | Glycocholic acid | 412,337,319,145 | ↑ | Primary bile acid biosynthesis |
| 45 | 9.72 | 299.1853 | -4.2 | C_15_H_26_O_3_ | 8-Hydroxy-4(6)-lactarene-5,14-diol | 281,237 | ↑ | - |
| 46 | 9.74 | 367.2107 | -5.1 | C_20_H_32_O_6_ | Prostaglandin G2 | 349,299,180 | ↑ | Arachidonic acid metabolism |

↑Compared with CON group, the level of metabolites in MOD group were significantly ncreased（*P*<0.05 or *P*<0.01）; ↓Compared with CON group, the level of metabolites in MOD group were significantly reduced (*P*<0.05 or *P*<0.01)

**TABLE S4** Characterization of components absorbed into blood from DSS in both positive and negative ion mode

| Rt/min | Element  composition | Adducts | Compound Name | PPM  ESI^-^/ESI^+^ | m/z  ESI^-^/ESI^+^ | MS/MS | Origin |
| --- | --- | --- | --- | --- | --- | --- | --- |
| 1.66 | C_13_H_16_O_9_ | M-H | Gentisic acid 5-O-glucoside | -1.4/— | 315.0716 | 153.0177,124.0184,109.0264 | RPA/CR/AR/AMR/ASR |
| 1.81 | C_15_H_14_O_6_ | M+H | Cianidanol | —/1.0 | 291.0866 | 139.0398,123.0413 | RPA/CR/P/AMR/ASR |
| 2.48 | C_16_H_18_O_9_ | M-H | Chlorogenic acid | -2.1/— | 353.0870 | 191.0557,135.0453,85.0289 | CR/P/AMR/ASR |
| 2.73 | C_16_H_26_O_8_ | M+Na | Jasminoside B | —/-0.7 | 369.1520 | 167.1076,111.0794,93.0681 | RPA |
| 3.68 | C_23_H_28_O_11_ | M-H/M+Na | Albiflorin | -1.5/-2.1 | 525.1603/481.1696 | 357.1206,121.0294/319.1172,197.0806,105.0312 | RPA |
| 4.09 | C_23_H_28_O_11_ | M+HCOO/M+Na | Paeoniflorin | 2.3/-1.9 | 525.1604/503.1519 | 479.1547,165.0551,121.0286/179.0694,151.0745 | RPA |
| 4.20 | C_9_H_10_O_5_ | M-H | Ethyl gallate | 1.1/— | 197.0457 | 169.0189,140.0112,124.0157 | RPA/CR/P/AMR/ASR |
| 4.60 | C_10_H_10_O_4_ | M-H/M+H-H_2_O | Ferulic acid | 0.2/-0.3 | 193.0506/177.0545 | 178.0277,134.0381,133.0298/149.0609,105.0315,77.0361 | ASR |
| 6.13 | C_12_H_16_O_4_ | M+H-H_2_O | Senkyunolide I | —/-0.8 | 207.1014 | 189.0936,161.0965 | CR/ASR |
| 6.78 | C_8_H_8_O_4_ | M+H-H_2_O | Griffonilide | —/-4.1 | 151.0382 | 105.0344,77.0402 | CR/ASR |
| 9.12 | C_12_H_14_O_3_ | M-H | 4-Hydroxy-3-(3-methylbut-2-enyl)benzoic acid | 0.1/— | 205.0870 | 161.0973,131.0507,106.0424 | RPA/CR/P/AR/ASR |
| 10.21 | C_12_H_16_O_2_ | M+H | 3-N-butyl-4,5-dihydrophthalide | —/-0.2 | 193.1222 | 175.1114,137.058 | CR/ASR |
| 10.41 | C_11_H_14_O | M+H-H_2_O | Isovalerophenone | —/-3.0 | 145.1006 | 130.0764,115.0524,77.0370 | RPA/CR/P/AMR/ASR |
| 10.42 | C_15_H_20_O_3_ | M+H-H_2_O | Atractylenolide III | —/0.4 | 231.1380 | 185.1305,161.0583,77.0375 | AMR |
| 11.07 | C_12_H_18_O_2_ | M+H | Sedanolide | —/-0.8 | 195.1377 | 149.1318,125.0576,79.0534 | All |
| 11.11 | C_12_H_14_O_2_ | M+H | Z-Ligustilide | —/-1.2 | 191.1064 | 173.0941,155.0839,145.0985 | CR/ASR |
| 11.18 | C_32_H_48_O_6_ | M+Na | Alisol C 23-acetate | —/2.7 | 551.3327 | 529.3499,469.3302,451.3188 | AR |
| 12.00 | C_30_H_50_O_5_ | M+HCOO | Alisol A | -2.4/— | 535.3628 | 395.2907,339.2697 | AR |
| 12.32 | C_31_H_48_O_4_ | M-H | Dehydrotumulosic acid | -1.8/— | 483.3470 | 439.3552,423.3223 | RPA/P/AMR |
| 12.63 | C_3_1H_46_O_5_ | M-H/M+H-H_2_O | Poricoic acid A | -2.2/-0.4 | 497.3261/481.3310 | 423.2900,379.3000/325.2124,307.2050 | P |
| 12.77 | C_30_H_48_O_5_ | M+Na | Alisol F | —/-1.6 | 511.3386 | 451.3169,433.3036 | AR |
| 14.01 | C_24_H_28_O_4_ | M+Na | Levistilide A | —/-1.4 | 403.1874 | 381.2104,191.1064,173.0949 | CR/ASR |
| 14.37 | C_32_H_50_O_5_ | M+Na | Alisol B acetate | —/-1.7 | 537.3543 | 497.3633,159.1179,121.0983 | AR |

Note: ASR:Angelicae Sinensis Radix; RPA:Radix Paeoniae Alba; RC:Chuanxiong Rhizoma; AR:Alismatis Rhizoma; P:Poria; AMR:Atractylodis Macrocephalae Rhizoma; ALL:ASR, RPA, CR, AR, P, AMR; √: Serum components.

**TABLE S5** The enrichment analysis of common target KEGG pathway based on the false discovery rate less than 0.05

| term description | matching proteins in your network (labels) | false discovery rate |
| --- | --- | --- |
| TNF signaling pathway | NFKB1,IL1B,PIK3CA,MMP14,PTGS2,TNF | 1.19E-06 |
| Estrogen signaling pathway | MMP2,PIK3CA,PGR,ESR2,BCL2,ESR1 | 2.55E-06 |
| Prolactin signaling pathway | NFKB1,PIK3CA,ESR2,CYP17A1,ESR1 | 3.44E-06 |
| NF-kappa B signaling pathway | NFKB1,IL1B,PTGS2,BCL2,TNF | 1.58E-05 |
| C-type lectin receptor signaling pathway | NFKB1,IL1B,PIK3CA,PTGS2,TNF | 1.58E-05 |
| IL-17 signaling pathway | NFKB1,IL1B,PTGS2,TNF | 0.00019 |
| Toll-like receptor signaling pathway | NFKB1,IL1B,PIK3CA,TNF | 0.0002 |
| Prostate cancer | NFKB1,PIK3CA,AR,BCL2 | 0.0002 |
| Sphingolipid signaling pathway | NFKB1,PIK3CA,BCL2,TNF | 0.00028 |
| Steroid hormone biosynthesis | CYP3A4,CYP17A1,CYP19A1 | 0.00074 |
| NOD-like receptor signaling pathway | NFKB1,IL1B,BCL2,TNF | 0.00082 |
| GnRH secretion | PIK3CA,CACNA1D,ESR2 | 0.00082 |
| Cortisol synthesis and secretion | CACNA1D,KCNK3,CYP17A1 | 0.00086 |
| Chemokine signaling pathway | NFKB1,PIK3CA,CCR1,JAK3 | 0.001 |
| Adipocytokine signaling pathway | NFKB1,PPARA,TNF | 0.001 |
| Calcium signaling pathway | CACNA1D,AVPR1A,PTGFR,CHRM5 | 0.0011 |
| cAMP signaling pathway | NFKB1,PIK3CA,CACNA1D,PPARA | 0.0013 |
| Longevity regulating pathway | NFKB1,PIK3CA,PPARG | 0.0016 |
| GnRH signaling pathway | MMP2,CACNA1D,MMP14 | 0.0017 |
| Inflammatory mediator regulation of TRP channels | IL1B,PIK3CA,TRPV4 | 0.002 |
| T cell receptor signaling pathway | NFKB1,PIK3CA,TNF | 0.0024 |
| HIF-1 signaling pathway | NFKB1,PIK3CA,BCL2 | 0.0026 |
| Neurotrophin signaling pathway | NFKB1,PIK3CA,BCL2 | 0.003 |
| MAPK signaling pathway | NFKB1,IL1B,CACNA1D,TNF | 0.0036 |
| Relaxin signaling pathway | MMP2,NFKB1,PIK3CA | 0.0041 |
| Phospholipase D signaling pathway | PIK3CA,AVPR1A,PTGFR | 0.0058 |
| PI3K-Akt signaling pathway | NFKB1,PIK3CA,BCL2,JAK3 | 0.0068 |
| JAK-STAT signaling pathway | PIK3CA,BCL2,JAK3 | 0.0069 |
| Transcriptional misregulation in cancer | NFKB1,PPARG,MEN1 | 0.0082 |
| VEGF signaling pathway | PIK3CA,PTGS2 | 0.013 |
| Arachidonic acid metabolism | PTGS1,PTGS2 | 0.0146 |
| Cytosolic DNA-sensing pathway | NFKB1,IL1B | 0.0149 |
| Fc epsilon RI signaling pathway | PIK3CA,TNF | 0.0165 |
| RIG-I-like receptor signaling pathway | NFKB1,TNF | 0.0179 |
| PPAR signaling pathway | PPARG,PPARA | 0.0197 |
| B cell receptor signaling pathway | NFKB1,PIK3CA | 0.0205 |
| Aldosterone synthesis and secretion | CACNA1D,KCNK3 | 0.027 |
| Thyroid hormone signaling pathway | PIK3CA,ESR1 | 0.0386 |
| AMPK signaling pathway | PIK3CA,PPARG | 0.0389 |
| Metabolic pathways | PIK3CA,CYP3A4,PTGS1,PTGS2,CYP17A1,CYP19A1 | 0.0433 |


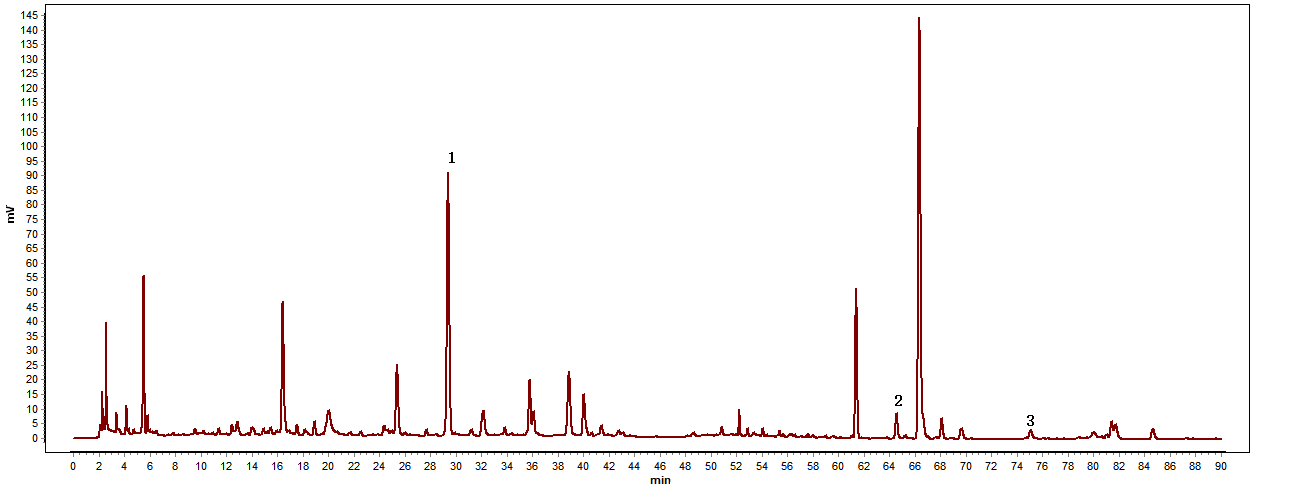


FIGURE S1 Chromatogram of alcohol extract from Danggui Shaoyao San. 1.Ferulic acid, 2. Alisol A, 3. Atractylenolide Ⅱ
